# A Century (1906‐2024) of Groundwater and Land Subsidence Studies in Greater Houston Region: A Review

**DOI:** 10.1111/gwat.70003

**Published:** 2025-07-09

**Authors:** Michael J. Turco, Ashley Greuter, Guoquan Wang

**Affiliations:** ^1^ Harris‐Galveston Subsidence District 1660 West Bay Area Blvd Friendswood TX 77546

## Abstract

The Greater Houston region has undergone substantial land subsidence over the past century, with rapid subsidence occurring from the late 1940s to the 1970s and more controlled rates thereafter. The establishment of the Harris‐Galveston Subsidence District (HGSD) in 1975 marked a pivotal milestone in subsidence management, primarily by regulating previously uncontrolled groundwater extraction. HGSD's success in reducing subsidence while simultaneously fostering robust economic growth in the Houston area inspired the creation of the Fort Bend Subsidence District (FBSD) in 1989. By 2024, significant subsidence (>0.3 m from 1906 to 2024) had impacted an area of approximately 12,000 km^2^, encompassing nearly all of Harris and Galveston Counties, as well as parts of the surrounding counties. This subsidence led to an irreversible loss of around 12 km^3^ of groundwater storage capacity—equivalent to 60 times the volume of Lake Houston, or roughly 8 years' worth of water usage for Harris and Galveston Counties as of 2023. About 65% of this loss occurred before HGSD regulations (1906‐1978), 20% between 1979 and 2000, and 15% since 2001. Due to groundwater regulations, the extent of subsidence has decreased significantly since the 1990s. By the early 2020s, the areas experiencing subsidence rates exceeding 1 cm/year had decreased to 1500 km^2^, roughly one‐twentieth of the greater Houston region, with only 50 km^2^ seeing rates above 2 cm/year. The highest current subsidence rate, approximately 3 cm/year since 2020, occurs in the Katy area, Fort Bend County. This review provides a comprehensive overview of land subsidence and groundwater level monitoring in the greater Houston region, highlighting regulatory developments, technological advancements, key research findings, and the continuing challenges of achieving sustainable groundwater management.

## Introduction

**Figure 1:**
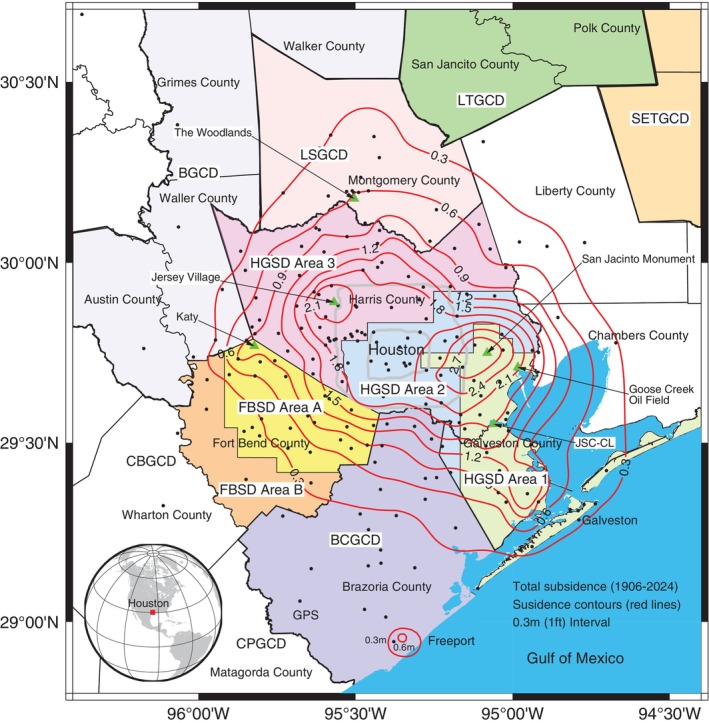
This map depicts the Greater Houston region, highlighting areas covered by subsidence and groundwater conservation districts. Key subsidence districts and groundwater conservation districts (GCDs) include: HGSD (Harris‐Galveston Subsidence District, Areas 1, 2, and 3); FBSD (Fort Bend Subsidence District, Areas A and B); BCGCD (Brazoria County GCD); CPGCD (Coastal Plains GCD); CBGCD (Coastal Bend GCD); BGCD (Bluebonnet GCD); LSGCD (Lone Star GCD); LTGCD (Lower Trinity GCD); and SETGCD (Southeast Texas GCD). The contour lines illustrate the cumulative land surface subsidence (in meters) from 1906 to 2024. The dark dots indicate the 220 reference sites used to generate the subsidence contours. The majority of reference sites coincide with GPS stations. Data sources and methodologies used to create the subsidence contours are described in the “Consequences of Permanent Subsidence: Groundwater Storage Capacity Loss” section of this paper.

The Greater Houston region, situated in southeastern Texas near the Gulf of Mexico, spans approximately 30,000 km^2^ and encompasses portions or all of about 15 counties, with Harris County at its center (Figure 1). This region has been one of the fastest‐growing metropolitan areas in the United States since the 2000s, boasting a population of over 7 million as of the early 2020s, according to the U.S. Census Bureau. The first land surface elevation survey in this region was conducted in 1905 and 1906 (Winslow and Doyel 1954). As of the 2020s, it has a history of about 120 years of land subsidence monitoring. The earliest groundwater‐level measurements in the Houston area date back to the 1890s. Beginning in the early 1930s, the U.S. Geological Survey (USGS), in partnership with the City of Houston and the Texas Water Commission, has systematically gathered groundwater‐level data, as well as information on groundwater pumpage and water quality, providing a comprehensive record of the changes and evolution of the region's groundwater resources (Wood and Gabrysch 1965).

Geologically, the region is predominantly composed of unconsolidated sediments from the Holocene and Miocene epochs, forming the extensive Gulf Coast aquifer system. This system, stretching from the Texas–Louisiana border to the Mexican border, is essential for the area's water resources (Casarez 2020). The Gulf Coast aquifer system comprises the Chicot, Evangeline, Burkeville confining unit, and Jasper aquifers (Baker Jr. 1979; Chowdhury and Turco 2006). Notably, the Chicot and Evangeline aquifers are hydrologically connected (e.g., Borrok and Broussard III 2016). Recently, the USGS has integrated these two aquifers into a single unit for groundwater‐level mapping purposes, now referred to as the Chicot‐Evangeline aquifer (undifferentiated) (Ramage et al. 2022). The Burkeville confining unit, located between the Evangeline and the deeper Jasper aquifer, serves as a regional stratigraphic barrier (e.g., Young and Draper 2020).

The earliest documented subsidence in the Greater Houston region occurred in the Goose Creek oil field near Baytown, approximately 40 km east of downtown Houston, in the early 1920s (Pratt and Johnson 1926). This event marked the beginning of the region's challenges with subsidence. The location of the Goose Creek oil field is marked in Figure 1. By 1926, geologists documented approximately 1 m of subsidence from 1917 to 1925, attributed to oil, gas, and groundwater extraction, causing significant portions of the field, originally on land, to become wholly or partially submerged in Tabbs Bay (Pratt and Johnson 1926). As of the late 1980s, the cumulative subsidence at the oil field approached approximately 2 m. Much of the field's productive area, particularly along the northern shoreline of Tabbs Bay, is now submerged or at sea level due to cumulative subsidence since the 1920s. Subsidence in the field area has stabilized since the 1990s due to reduced groundwater and hydrocarbon withdrawals, but the submerged portions remain underwater, contributing to local flooding risks and wetland loss. From the late 1940s to the mid‐1970s, rapid subsidence, occurring at rates of up to a decimeter per year, became increasingly pronounced in the southeastern parts of Houston, particularly in areas such as Baytown, Pasadena, Clear Lake (CL), and along the Houston Ship Channel and Galveston Bay. Declining groundwater levels (GWLs), driven by rapid industrial expansion, resulted in over 2 m of subsidence in the area along the Houston Ship Channel between Baytown and Houston from the 1940s to the mid‐1970s. By 1979, as much as 3 m of subsidence had been documented in the Baytown area, covering more than 8000 km^2^ with at least 0.3 m of subsidence (Gabrysch 1982). As the population expanded to the north and northwest from Houston since the 1990s, groundwater pumping and consequent land subsidence followed this urban growth trend, affecting areas around Katy, Jersey Village, and The Woodlands with moderate subsidence rates of 1 to 3 cm per year as of the early 2020s.

The dynamic interplay between groundwater resource management, subsidence, urban development, and environmental sustainability has been central to the Greater Houston region's approach to addressing these critical issues. This paper delves into how regulatory initiatives and technological advancements have influenced current practices and policies, aiming to strike a balance between development needs and the reduction in groundwater use to prevent subsidence.

## A Brief History of Subsidence and Groundwater Regulations

### Subsidence Processes and Aquifer Consolidation Cycles

The history of land surface elevation monitoring in the Greater Houston region spans over a century, from the 1900s to the 2020s. Fundamentally, land subsidence results from the compaction of aquifers, with inelastic compaction occurred in clays leading to permanent subsidence. The principle of land subsidence is governed by Terzaghi's Soil Consolidation Theory (Terzaghi 1925), which describes how changes in pore water pressure within aquifer systems drive sediment compaction. This compaction process, known as aquifer consolidation, is initiated by excessive groundwater pumping, which reduces pore water pressure, increases effective stress, and leads to compaction of sediments, primarily sand and clay, resulting in land subsidence. According to Terzaghi's theory, the effective stress (σ') (exerted on the sediment matrix of the aquifer) can be defined as $\sigma^{\prime }=\sigma -\mu$, where σ is the total stress and μ is the pore water pressure. As pumping lowers μ, $\sigma^{\prime }$ increases, leading to compaction of the sediment matrix. Aquifer consolidation involves a cycle that begins when GWLs drop below the historical pre‐consolidation head, initiating inelastic compaction. Subsidence continues until pore water pressure within the aquifer system rises to a new equilibrium, ultimately leading to the cessation of permanent subsidence and, in some cases, the initiation of minor land rebound (Wang 2023a).

**Figure 2:**
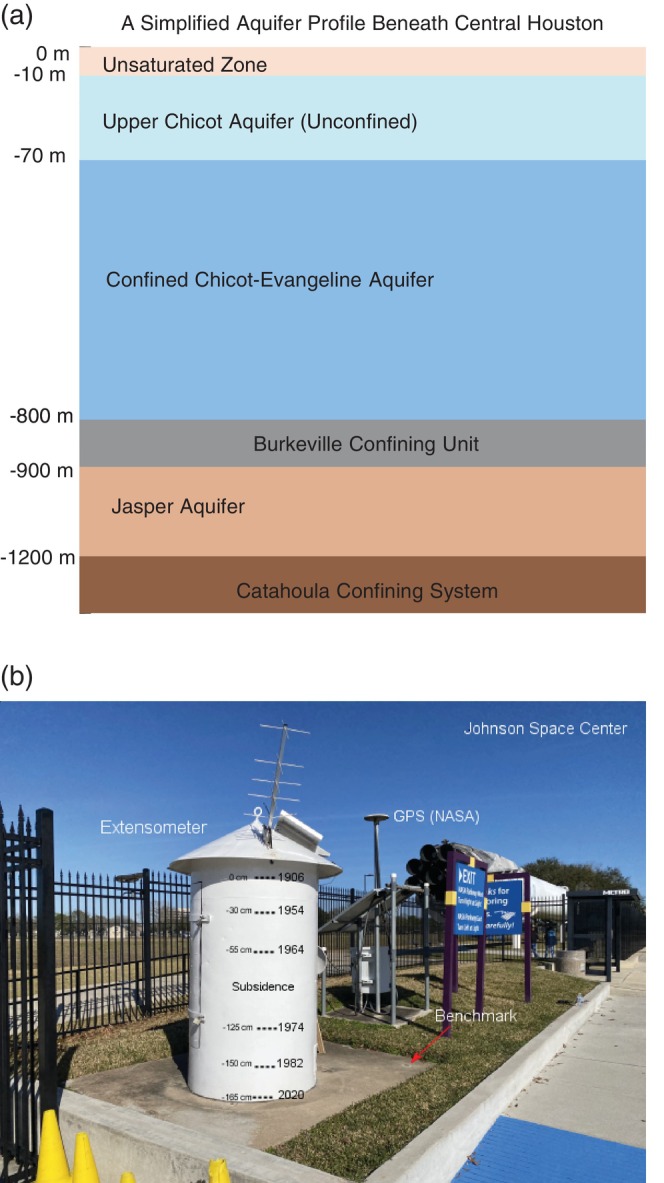
(a) An illustration depicting the aquifer system beneath the Johnson Space Center (JSC) and Clear Lake (CL) area, indicating the depths of three extensometers and the long‐standing groundwater monitoring well KH‐65‐32‐714 (1931‐1994, Well depth: −170 m). Note the schematic is not drawn to scale and is intended for visual purposes only. (b) A photograph at the JSC monitoring site, featuring a permanent benchmark, a borehole extensometer (1962‐2024), and a GPS station (NASA, 2014‐2024). In the JSC‐CL area, three borehole extensometers are present: Clear Lake shallow extensometer (borehole depth: −530 m), Clear Lake deep extensometer (−936 m), and Johnson Space Center extensometer (−235 m). Each extensometer borehole also functions as a piezometer measuring hydraulic head in the confined Chicot‐Evangeline aquifer.

The consolidation cycle in the Houston area can be exemplified by long‐term observations of land surface elevation and GWL changes around the Johnson Space Center (JSC) and CL, situated on the boundary between Harris and Galveston counties. The location of the JSC‐CL area is marked in Figure 1. Figure 2a illustrates the aquifer system beneath the JSC‐CL area, highlighting the depths of an extensometer at JSC and two nearby extensometers in the CL area. The upper sediments, at depths shallower than approximately 70 m (or 200 ft), are considered unconfined aquifers (Ramage et al. 2022). Figure 2b showcases the subsidence monitoring infrastructure at the entrance of the JSC, including a borehole extensometer and a Global Positioning System (GPS) station, both essential for subsidence monitoring. The JSC extensometer, operated by the USGS since 1962, works in conjunction with a permanent GPS station operated by the University of Houston (UH) since 2014. Additionally, a National Geodetic Survey (NGS) benchmark for vertical‐control surveying is also located nearby.

**Figure 3:**
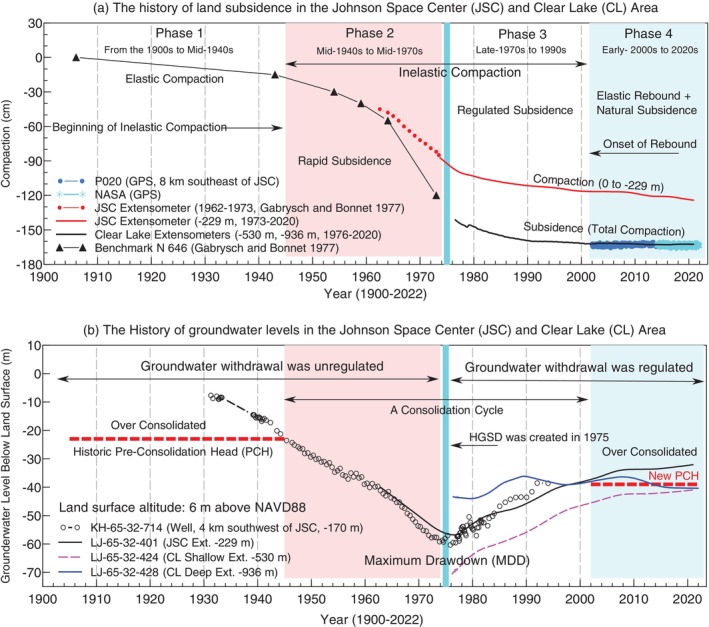
Historical trends of land subsidence and groundwater levels in the confined Chicot‐Evangeline aquifer system beneath the Johnson Space Center (JSC) and Clear Lake (CL) area. (a) Aquifer compaction from 0 to 229 m below land surface, recorded by the JSC extensometer, and land subsidence (total aquifer compaction) in the JSC‐CL area, recorded by the CL extensometers, from the 1900s to the 2020s. (b) Groundwater levels in the confined Chicot‐Evangeline aquifer beneath the JSC‐CL area from the 1930s to the 2020s. In this area, the confined aquifer system experienced its maximum drawdown (MDD) in the mid‐1970s. A profile of the aquifer system and a site photo of the JSC extensometer are shown in Figure 2.

Figure 3a illustrates the history of aquifer compaction and total land subsidence (1906‐2024) in the JSC‐CL area, while Figure 3b presents the historical GWLs within the confined Chicot‐Evangeline aquifer, the primary freshwater source for this region. The JSC extensometer (operating from 1962 to 2024), which reaches 229 m below the land surface (abbreviated as −229 m), has been essential for measuring sediment compaction within this depth range. Additional comprehensive data on total compaction were gathered from a NGS first‐order benchmark (N 646) and two other extensometers located in CL, about 2.5 km from the JSC site. In 1976, the USGS installed two closely spaced extensometers at the CL site, extending to depths of 530 and 936 m. The shallow borehole was completed in the midsection of the Chicot‐Evangeline aquifer, while the deep borehole extended to its base, resting on top of the Burkeville confining unit (see Figure 2a). The shallow and deep extensometers recorded nearly identical vertical displacements throughout their entire monitoring period (Figure 3a), indicating no significant aquifer compaction below the depth of the shallow borehole at −530 m.

While subsidence patterns vary across the Houston region, the evolution of subsidence, or the consolidation cycle, can be divided into four distinct phases (Figure 3a): (1) the pre‐ to early‐development years before the mid‐1940s, (2) the rapid subsidence era from the mid‐1940s to the mid‐1970s, (3) the era of regulated subsidence from the late‐1970s to the late‐1990s, and (4) the period of stabilized subsidence ongoing since the 2000s. In Phase 1, GWLs gradually declined but remained above the historical pre‐consolidation head of the confined Chicot‐Evangeline aquifer. The primary mechanism of aquifer compaction was elastic deformation, characterized by a strain‐to‐stress ratio typically between 1:500 and 1:1000 within the Houston area. This range suggests that a 5 to 10‐m drop in GWLs could yield approximately 1 cm of elastic compaction (recoverable), with variations influenced by the thickness of sand layers in the aquifers, the depth of groundwater extraction, and other factors (Wang 2023b).

Phase 2 represented a transformative period as GWLs in the confined Chicot‐Evangeline aquifer fell below the historical pre‐consolidation head, reaching about 25 m below the land surface in the JSC‐CL area by the early 1940s. This substantial decline initiated inelastic compaction (unrecoverable), which led to permanent subsidence. The inelastic strain‐to‐stress ratio during this phase was significantly higher, about 1:50 to 1:100, indicating a more dramatic compaction compared to the elastic phase (Wang 2023b).

Phase 3 marked an era of transition, fueled by groundwater regulation efforts that began raising GWLs. This led to a gradual reduction in the rate of land subsidence, from several centimeters per year initially to a few centimeters, and eventually to less than 1 cm/year. Notably, subsidence in the JSC‐CL area ceased in the 1990s, although GWLs started to rise shortly after regulations were enacted in the mid‐1970s, showcasing a pronounced delay in the response of subsidence cessation.

Phase 4 began with the onset of land rebound, marked by an equilibrium in pore water pressure across the various sand and clay layers distributed throughout the confined aquifer system. During this phase, GWLs not only reached but also exceeded the new pre‐consolidation head (NPCH), which serves as a critical threshold to prevent the reoccurrence of permanent subsidence (Wang 2023a). As long as GWLs are maintained above the NPCH, the risk of further permanent subsidence is mitigated. Elastic expansion, which primarily occurs in sand layers as GWLs rise, is expected to continue for many years to even decades. With the cessation of anthropogenic subsidence, natural subsidence processes, which are estimated to contribute about 2 mm/year subsidence in the JSC‐CL area, have become more pronounced and easier to observe (Zhou et al. 2021).

### Development of Groundwater Regulations

Founded in 1837, the city of Houston initially relied on groundwater as its primary water resource. The first wells, approximately 40 to 50 m deep, were established in the downtown area in the late 1890s, where the groundwater was found to be free flowing (Wood and Gabrysch 1965). Historically, GWLs in the confined Chicot‐Evangeline aquifer across the Houston area remained above the land surface until the early 1910s. A significant shift in groundwater extraction occurred with the onset of industrial pumping from deep wells, following the opening of the Houston Ship Channel in late 1914.

#### Harris‐Galveston Subsidence District (HGSD)

Since the early 1900s, the majority of groundwater withdrawals in the Houston region have been sourced from the shallow portion of the Chicot‐Evangeline aquifer (e.g., shallower than 150 m below land surface), with recent expansions reaching into the deep portion of the Chicot‐Evangeline aquifer and its underlay Jasper aquifer, especially in the northern Houston area. Concerns regarding Houston's dependency on groundwater emerged significantly in the 1960s, as extensive pumping efforts led to rapid land subsidence, ranging from several centimeters to a decimeter per year. This subsidence resulted in frequent damage to infrastructure, including buildings, roadways, bridges, underground utility lines, and levees, and increased the risk of flooding. To address the subsidence issue, the 64th Texas Legislature established the HGSD in 1975, marking the first such district in the United States. Since its inception, the HGSD has enacted groundwater regulations aimed at reducing groundwater pumping.

**Figure 4:**
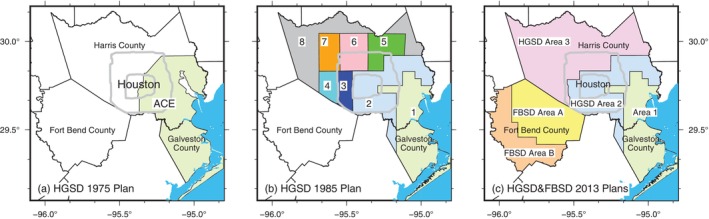
The evolution of groundwater regulation plans implemented by the Harris‐Galveston Subsidence District (HGSD) over time. (a) The initial plan, introduced in 1975, regulated groundwater pumpage in all of Galveston County and much of southeastern Harris County, designated as the Area of Concentrated Emphasis (ACE). (b) The 1985 updated plan divided Harris and Galveston Counties into eight zones, with further adjustments made in 1992 and 1999. (c) The current HGSD plan, in effect since 2013 and amended on April 14, 2021, is shown alongside the groundwater regulation plan implemented by the Fort Bend Subsidence District (FBSD) since 2013.

The inaugural HGSD groundwater regulatory plan was formulated in 1975 and implemented in 1976, undergoing subsequent updates in 1985, 1992, 1999, and 2013. The initial plan prioritized regions undergoing significant subsidence and having access to alternative surface water sources. It established regulations on groundwater pumpage throughout most of Galveston County and large parts of southeastern Harris County, within a designated zone called the “Area of Concentrated Emphasis” (ACE) (Figure 4a). The 1985 adjustments led to the segmentation of HGSD's jurisdiction into eight regulatory zones (Figure 4b), with an emphasis on gradually transitioning to alternative water resources, primarily surface water. This division was further refined in the 1992 plan, which consolidated previous six zones (from Zone 3 to Zone 8) illustrated in Figure 4b into five zones. The 1999 plan further simplified the region into three areas by merging the northern and western Harris County into a single zone, known as Area 3. The 2013 Regulatory Plan continues to categorize Harris and Galveston counties into three regulatory areas: Area 1, Area 2, (HGSD 2013) and Area 3, as shown in Figure 4c. Each regulatory plan has been guided by observations of groundwater and subsidence patterns, alongside projections concerning water demand, population growth, and economic development.

The regulatory plan sets an annual groundwater withdrawal limit for users based on their geographic location, calculated as a percentage of their total water demand, with the goal of gradually reducing groundwater dependency. The current HGSD regulatory plan aims to restrict groundwater withdrawal to no more than 10% of the total water demand in Area 1 and no more than 20% in Area 2. These targets have been met since 2000, with a few exceptions in the eastern part of Area 2. In Area 3, groundwater users that are not operating under a certified groundwater reduction plan (GRP) are permitted to withdraw up to 20% of their annual water demand. Currently, from 2010 to 2024, those with a certified GRP can withdraw up to 70% of their water demand from groundwater, but this will decrease to 40% starting in 2025 and further reduce to 20% beginning in 2035 (HGSD 2013).

HGSD's groundwater regulations have significantly reduced the rates of subsidence in Areas 1 and 2 shortly after the implementation of the initial groundwater regulatory plan. By the early 1990s, subsidence in southeastern Houston and Galveston (Areas 1 and 2) had effectively ceased. The rate and extent of subsidence within the HGSD jurisdiction have steadily declined since the implementation of groundwater regulations in HGSD Area 3 in 2010 (e.g., Turco and Petrov 2015). Yet, since the mid‐1900s, subsidence began to spread to the northern and western regions, primarily within HGSD Area 3, northern Fort Bend County, and southern Montgomery County, mirroring the pattern of urban expansion from coastal to inland areas.

**Figure 5:**
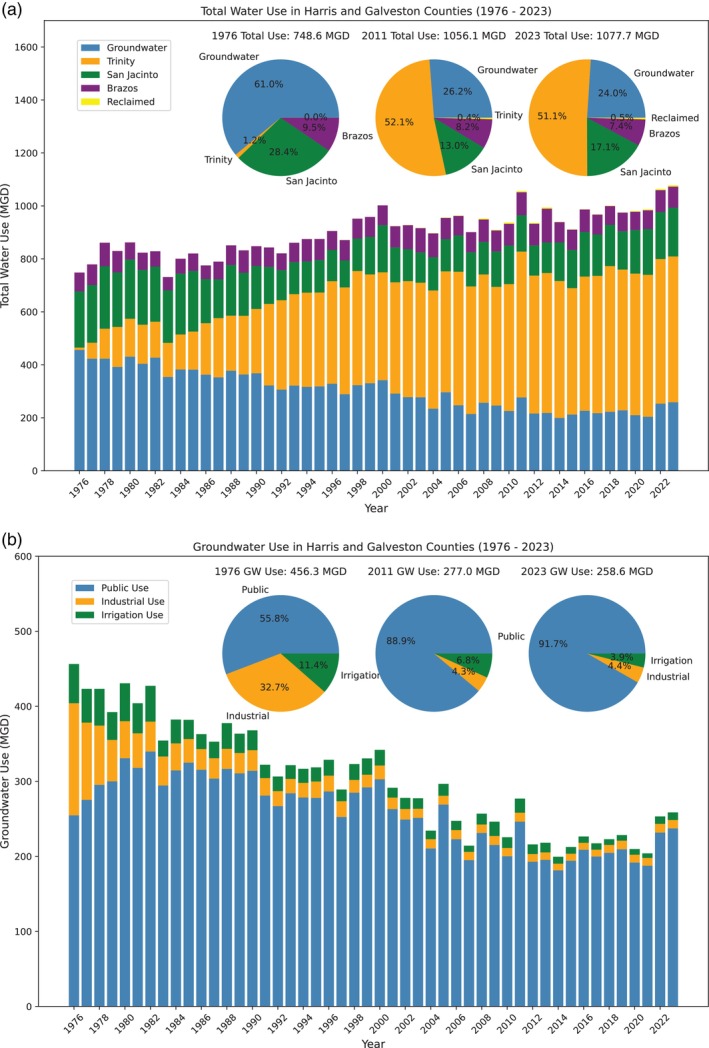
(a) Yearly total water usage in Million Gallons per Day (MGD) within the HGSD jurisdictional area, encompassing Harris and Galveston Counties, from 1976 to 2023, primarily sourced from groundwater, Trinity River, San Jacinto River, Brazos River, and reclaimed water. (b) Annual groundwater withdrawal volume within the HGSD jurisdictional area, encompassing Harris and Galveston Counties, from 1976 to 2023, categorized by primary usage in public, industrial, and irrigation sectors.

Since 1976, water users within the HGSD jurisdiction have increasingly transitioned from groundwater to alternative sources, primarily treated surface water, in accordance with the HGSD's regulatory plan. The percentage of total water demand met by groundwater has significantly declined within the HGSD jurisdiction, from approximately 61% in 1976 to about 24% by 2023 (Figure 5a). As of 2023, which marked a second year of exceptional drought conditions, groundwater withdrawals within the HGSD jurisdictional region are estimated at around 258.6 million gallons per day (MGD) (Figure 5b). Of this, 85% is extracted from Area 3, 12% from Area 2, and only 3% from Area 1 (Greuter 2024). As of 2023, groundwater is primarily used for public water supply, accounting for roughly 92% of total pumping, while industrial use and irrigation, which include agricultural and other irrigation, represent about 4% each (Figure 5b). Overall water usage within the HGSD jurisdiction has risen from approximately 748.6 MGD in 1976 to 1077.7 MGD in 2023, while reliance on groundwater has decreased from about 456.3 MGD to 258.6 MGD. This reduction in groundwater use was achieved through the continuation and expansion of alternative water sources. Surface water, sourced from the Trinity River, San Jacinto River, and Brazos River, provides approximately 75% of the region's water needs. Meanwhile, reclaimed water constitutes less than 1% of total water usage (Figure 5a).

Building on HGSD's success in reducing subsidence in Galveston County and parts of eastern Harris County, the Texas Legislature established the Fort Bend Subsidence District (FBSD) in 1989. Additionally, seven additional groundwater conservation districts (GCDs) were formed across the Greater Houston region to manage water resources and control subsidence (see Figure 1). These include the Lone Star GCD (LSGCD), established in 2001, serving Montgomery County; the Bluebonnet GCD (BGCD), created in 2001, responsible for Austin, Grimes, Walker, and Waller counties; the Coastal Bend GCD (CBGCD), established in 2002, overseeing Wharton County; the Coastal Plains GCD (CPGCD), created in 2001, managing Matagorda County; the Brazoria County GCD (BCGCD), formed in 2005, serving Brazoria County; the Southeast Texas GCD (SETGCD), established in 2004, responsible for Tyler, Hardin, Jasper, and Newton counties; and the Lower Trinity GCD (LTGCD), created in 2005, overseeing Polk and San Jacinto counties. As of 2024, Liberty and Chambers Counties remain the only neighboring counties to Harris and Galveston that have yet to implement groundwater management strategies.

The creation of these subsidence and GCDs marked a significant move toward adopting more sustainable groundwater management practices in the area. This effort required substantial coordination with regional and local ground and surface water providers, continuous collaboration with state and local regulatory agencies, and implementation of various water management strategies based on historical and projected water use.

#### Fort Bend Subsidence District

Established in 1989, the FBSD addresses subsidence concerns in Fort Bend County through the regulation of groundwater withdrawals. Its foremost objective is to regulate groundwater extraction to minimize subsidence through the implementation of its regulatory plan. To achieve this goal, FBSD has implemented various strategies, including the promotion of alternative water sources and encouraging a sense of collective responsibility among groundwater users. Echoing the HGSD's method, FBSD takes a flexible approach to groundwater regulation, carefully balancing the need for community and economic development while recognizing that excessively stringent regulations could hinder such progress.

Since its founding, FBSD has invested in comprehensive monitoring stations for GWLs and land surface elevations, continually refining groundwater and subsidence models to guide regulatory decisions. The initial regulatory plan, introduced in September 1990, highlighted the necessity for improved data collection on GWL and subsidence monitoring within the county. This regulatory plan saw updates in 2003 and 2013, with the latter being amended on June 21, 2022, which extended conversion timelines. The current 2013 Regulatory Plan divides Fort Bend County into two areas (Areas A and B) for groundwater regulation purposes (see Figures 1 and 4c). Water users located in Area A can use up to 40% of groundwater from their total water demand unless operating under a certified GRP. Groundwater users operating under a certified GRP shall not surpass more than 70% of groundwater from the total water demand (FBSD 2013). As of 2024, Area B is not subject to groundwater withdrawal reduction requirements.

**Figure 6:**
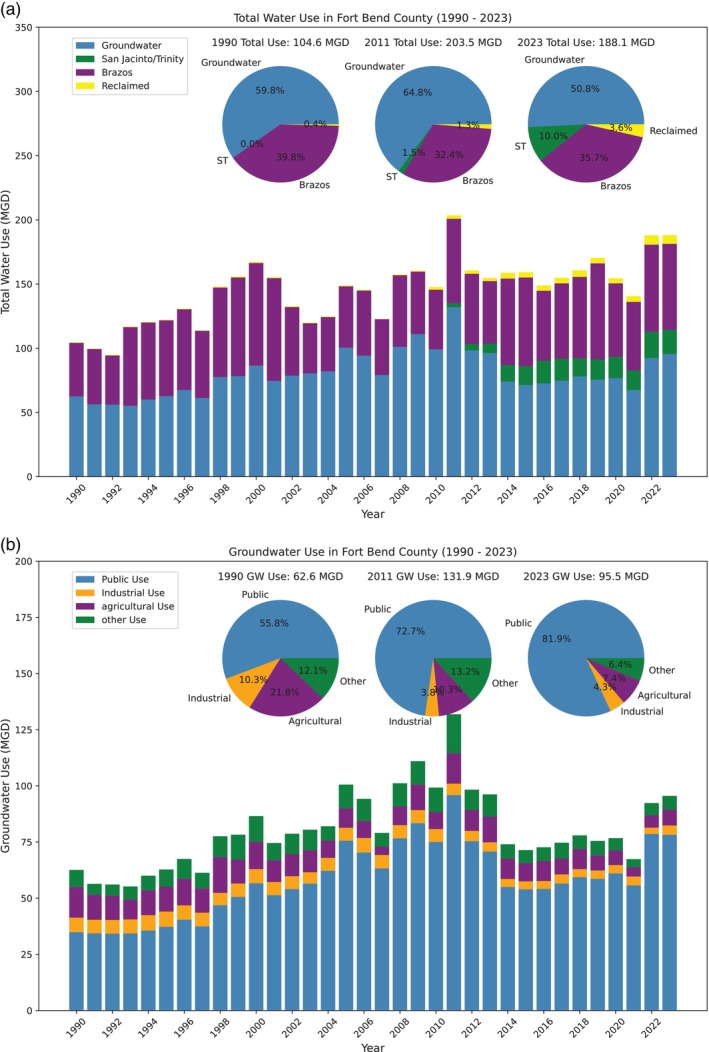
(a) Yearly total water usage in Million Gallons per Day (MGD) within the Fort Bend Subsidence District (FBSD) jurisdictional area, encompassing Fort Bend County, from 1990 to 2023, primarily sourced from groundwater, Brazos River, San Jacinto River, and Trinity River (ST), and reclaimed water. (b) Annual groundwater withdrawal volume within the FBSD jurisdictional area from 1990 to 2023, categorized by primary usage in public, industrial, agricultural, and other sectors.

Over the past three decades, total water demand in Fort Bend County has gradually increased alongside population growth, rising from around 100 MGD in the early 1990s to approximately 150 MGD since the 2010s. Water usage spiked above 200 MGD during the extreme drought of 2011, closely approaching 190 MGD during the drought periods of 2022 and 2023 (Figure 6a). As of 2023, groundwater accounts for approximately 51% of total water usage (188.1 MGD) in Fort Bend County, down from about 60% in the 1990s. Annual groundwater use was around 60 MGD in the 1990s, gradually increasing to roughly 100 MGD by the 2000s. Following the implementation of groundwater regulations in 2013, usage began to decline, dropping to 67 MGD by 2021. This trend reversed in 2022 and 2023 due to severe droughts during both summers, leading to a significant increase in groundwater use, which reached approximately 95 MGD in 2022 and 2023 (Figure 6b). Of this total, approximately 82% is allocated for public use, 8% for agriculture, 4% for industrial purposes, and 6% for other uses, such as outdoor irrigation and pond filling.

#### Groundwater Conservation Districts

Texas was divided into 16 groundwater management areas (GMAs), established during the 79th Texas Legislative Session in 2005. The majority of the Greater Houston region falls within GMA 14 (GMA14). The Wharton and Matagorda counties fall within GMA 15 (GMA15). The Greater Houston region comprises seven GCDs including five GCDs in GMA14 and two GCDs in GMA15 (Figure 1). Each GCD has its unique mission and regulations designed to ensure a sustainable future, emphasizing water resource security and land stability.

Each GCD enforces a comprehensive set of groundwater management plans and initiatives promoting responsible groundwater conservation and ensuring the long‐term availability of groundwater reserves for future generations. These measures include permitting protocols, continuous water level monitoring, conservation campaigns, and educational outreach efforts. Effective groundwater management relies on implementing policies that not only aim to reduce groundwater extraction rates but also encourage the use of alternative water sources, such as surface water, alongside the adoption of water conservation practices. Additionally, it requires ongoing monitoring of GWLs and modeling of potential subsidence, supplemented by research efforts to understand the dynamic responses of aquifers and land surfaces to management interventions.

## Groundwater‐Level Monitoring: Historical Overview and Decadal Maps (1920s‐2020s)

GWL monitoring in the Houston area boasts a long history, dating back to the 1890s. Prior to the 1900s, GWLs within the confined Chicot‐Evangeline aquifer were much higher than the land surface. Since 1977, the USGS has published annual reports that document both short‐term fluctuations and long‐term trends in GWLs within the Chicot and Evangeline aquifers beneath the Houston area, providing valuable insights into the region's hydrological dynamics (e.g., Gabrysch 1979; Barbie et al. 1991). In 2001, the USGS introduced the inaugural GWL map for the Jasper aquifer (Coplin 2001). Since then, the USGS has annually reported on GWL changes within the Jasper aquifer beneath Montgomery County and northern Harris County (e.g., Kasmarek et al. 2015; Ramage 2024).

**Figure 7:**
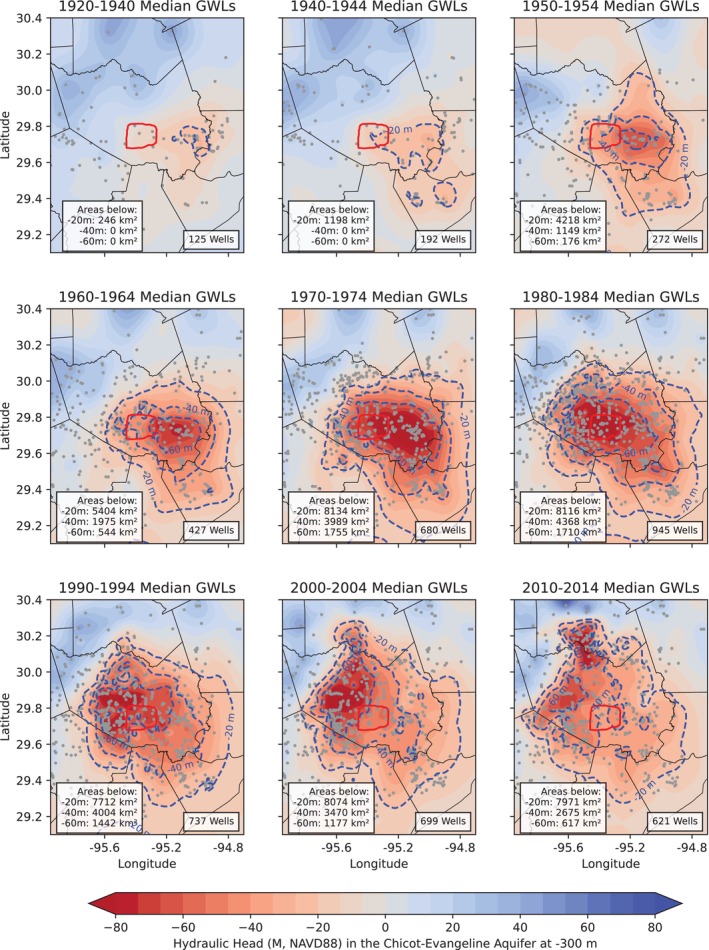
Groundwater levels (GWLs) in the confined Chicot‐Evangeline aquifer system. The first subfigure represents combined data from the 1920s to the 1930s, while the subsequent subfigures show data at the beginning of each decade from the 1940s to the 2010s. In each panel, the red enclosed area represents the Houston Loop 610, and gray dots mark well locations used for contour generation. Wells shallower than 70 m below‐ground surface were excluded. For each well, the GWL is represented by the median of all available measurements from the first 5 years of each decade and adjusted to a depth of 300 m below land surface using an intrinsic vertical hydraulic gradient (IVHG) of 0.07.

As of the early 2020s, the USGS routinely measures GWLs in about 650 wells across the primary aquifers, including the Chicot‐Evangeline aquifer, Burkeville confining unit, and Jasper aquifer. The comprehensive collection of GWL data by the USGS is publicly available through the USGS National Water Information System (NWIS) (USGS 2024). Figure 7 presents GWL contour maps of the confined Chicot‐Evangeline aquifer, showing changes at the start of each decade from the 1920s through the 2010s. For each well, the GWL is represented by the median of all available measurements from the first 5 years of each decade and adjusted to a standardized depth of 300 m below the land surface. This adjustment uses a vertical hydraulic gradient (VHG) of 0.07, meaning the hydraulic head decreases by approximately 7 m per 100 m increase in well depth. The VHG of 0.07 was derived from comprehensive GWL measurements in the Houston region during the 1920s to 1930s, when pore water pressure in the confined Chicot‐Evangeline aquifer was in equilibrium and no subsidence occurred, and from the recent data (2011‐2023) in the HGSD Regulatory Areas 1 and 2, where subsidence has ceased and pore water pressure has remained in equilibrium for approximately three decades (from the 1990s to 2010s). During periods of rapid subsidence, the VHG was higher than 0.07. The VHG of 0.07 corresponds to the intrinsic vertical hydraulic gradient (IVHG), representing the minimum gradient within the confined Chicot‐Evangeline aquifer system under near‐undisturbed, natural conditions (Wang 2025). The occurrence of IVHG is primarily due to the presence of numerous discontinuous clay layers or fine‐grained beds, which create resistance to vertical flow. Variations in hydraulic conductivity and permeability within the aquifer system also contribute to this minimal VHG. Additionally, factors like temperature and salinity variations may cause density‐driven vertical flows, further influencing the VHG.

The 300‐m depth represents the middle section of the “deformed” portion of the confined aquifer, which ranges from −100 to −600 m and has experienced the most intensive pumping activity and inelastic compaction. Kriging was applied to grid the GWL datasets, which assigns spatially dependent weights to measured values, allowing for more accurate predictions at unmeasured locations. Kriging has been widely recognized as an effective technique for interpolating hydrologic data, especially water‐level information (Varouchakis et al. 2012; Ramage et al. 2022).

**Figure 8:**
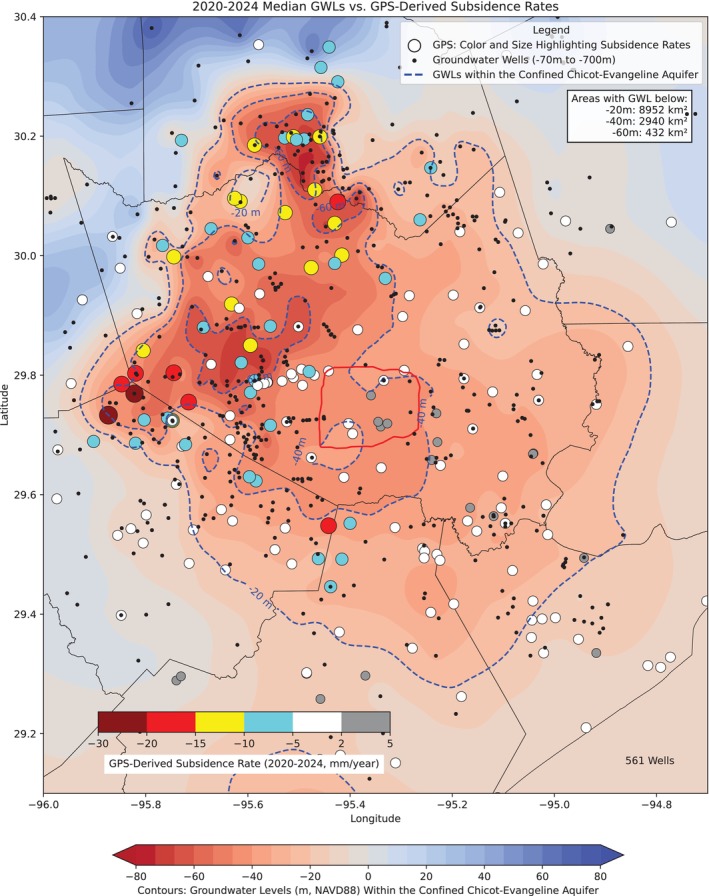
Map displaying present‐day (early 2020s) groundwater levels within the confined Chicot‐Evangeline aquifer, alongside land subsidence rates recorded at HoustonNet GPS sites. Groundwater levels at each well are represented by the median of readings from 2020 to 2024, standardized to a depth of 300 m below land surface using an Intrinsic Vertical Hydraulic Gradient (IVHG) of 0.07. Subsidence rates at each GPS site are estimated through linear regression of available measurements from 2020 to 2024. The red enclosed area represents the Houston Loop 610.

The delineation of the −40 and −60 m GWL contours vividly demonstrates the spread of groundwater pumping over time. The areas with the deepest GWL, enclosed by the −60 m contour lines, reached their maximum extent in the 1970s and 1980s. By the 2010s, the total area with GWL below −40 m had decreased to approximately 2675 km^2^, down from a peak of 4368 km^2^ in the 1980s. Similarly, the area with GWL below −60 m had shrunk to around 617 km^2^, compared to its maximum extent of 1755 km^2^ in the 1970s. Figure 8 displays a similar map of GWL as of the early 2020s, overlaid with the ongoing land subsidence rates (2000–2024) at HoustonNet GPS sites. By the early 2020s, the areas with GWLs below −40 and −60 m had reduced to approximately 2940 and 432 km^2^, respectively. Notably, the area with GWLs below −60 m is now smaller than it was in the early 1960s, when rapid subsidence in Houston began, at that time covering approximately 544 km^2^. There is a slight trend indicating an expansion in the areas with GWLs below −20 and −40 m compared to the early 2010s. This increase is likely due to heightened groundwater pumping during the severe droughts of 2022 and 2023. Groundwater use data, as shown in Figures 5 and 6, clearly indicate a significant rise in groundwater pumping during these drought years compared to previous years.

## Land Subsidence Monitoring: Established Methods and Current Rates (2014‐2024)

Monitoring changes in land surface elevation is essential for evaluating land subsidence (Galloway and Burbey 2011). Historically, subsidence was tracked through repeated spirit‐leveling surveys and extensometers prior to the 1990s. However, the advent of GPS technology in the late 1980s led to a gradual shift away from traditional leveling methods. By the mid‐1990s, GPS had emerged as the dominant tool for subsidence monitoring in the Houston region, offering greater precision and efficiency compared to conventional techniques. Despite their effectiveness, these earlier methods often produced data that was spatially sparse. To overcome this limitation, Interferometric Synthetic Aperture Radar (InSAR) has been increasingly adopted for subsidence studies since the 2000s, providing more spatially detailed measurements. Long‐term extensometer and tide gauge measurements have also been utilized to derive land subsidence.

**Figure 9:**
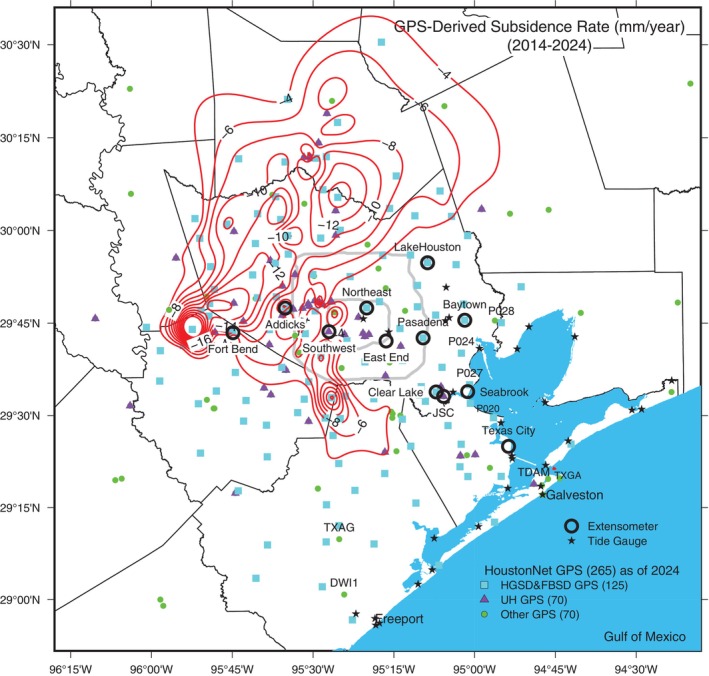
Map depicting subsidence monitoring stations in the Greater Houston region along with current subsidence‐rate contours (2014‐2024). The sites include about 265 GPS, 14 extensometers, and about 30 tide gauges. Subsidence rates are primarily based on GPS measurements collected over the past decade (2014‐2024).

Figure 9 shows the locations of GPS, extensometer, and tide gauge sites used to monitor land subsidence in the Greater Houston region, along with current subsidence rate contours primarily derived from GPS observations over the past decade (2014‐2024). A linear regression is applied to the vertical displacement time series (with a minimum duration of 3 years) to calculate the subsidence rate at each GPS site. By the early 2020s, approximately 5464 km^2^ experience subsidence rates exceeding 5 mm/year, and around 1500 km^2^ experience subsidence rates over 10 mm/year. Generally, subsidence with a rate below 5 mm/year is insufficient to cause significant infrastructural damage over a decade. Only around 50 km^2^, primarily located in the Katy area in Fort Bend County, near the southeast corner of the I‐10 and Highway 99 intersection, experience subsidence rates exceeding 20 mm/year over the past decade (2014‐2024).

### Leveling Survey

Spirit leveling is a highly accurate method for obtaining precise land surface elevations, thereby tracking changes over time. It is commonly used along roads, railroad tracks, aqueducts, and canals. The NGS and its predecessor agency, the U.S. Coast and Geodetic Survey, have established extensive networks of first‐ and second‐order level lines across the Houston area since the early 1900s. The region's first leveling survey, a first‐order line from Smithville to Galveston, was conducted in 1905 and 1906 (Winslow and Doyel 1954). In the decades that followed, this first‐order line was resurveyed multiple times, with additional second‐order survey lines added and repeatedly surveyed. The results derived from these leveling surveys have been published in a series of USGS reports. Gabrysch and Bonnet (1977) published subsidence contour maps for the periods 1906‐1943, 1943‐1974, and 1964‐1976; Gabrysch (1982) followed with contour maps for 1906‐1978, 1943‐1978, and 1973‐1978; and Gabrysch and Coplin (1990) presented maps for the spans 1906‐1987, 1943‐1987, 1978‐1987, 1978‐1983, and 1983‐1987. These subsidence contour maps offer crucial insights into the spatial progression of historical land subsidence in the Houston area, making them an essential resource for planning and mitigating subsidence‐related hazards.

### Continuous GPS Monitoring

Houston has been a pioneer in employing GPS technology for subsidence monitoring. In the late 1980s, before the completion of the GPS satellite constellation, campaign GPS surveys had been conducted to monitor subsidence at benchmarks throughout the region. In 1987, the HGSD installed a network of 82 benchmarks throughout the Houston area to monitor land subsidence using GPS technology. This benchmark network was resurveyed by using GPS in 1995, 2000, 2007, and most recently in 2022 (HGSD 2022). Using all available benchmark data, the USGS published subsidence contour maps for the periods 1906 to 1995 (Stork and Sneed 2002) and 1906 to 2000 (Gabrysch and Neighbors 2005). The HGSD further extended the total subsidence map through 2020 by incorporating all available GPS measurements since 2000 (Greuter et al. 2021).

Since the mid‐1990s, permanent GPS stations have become the primary tool for monitoring subsidence in Houston. The HGSD initiated the deployment of a GPS network, known as Port‐A‐Measure (PAM) stations, with the first PAM station operating in 1994 (Zilkoski et al. 2001). By 2024, the PAM network had grown to approximately 114 permanent GPS stations, with HGSD and FBSD continuously adding new stations in areas of interest.

Since the 2010s, the HGSD, FBSD, UH, and various local organizations have collaborated to establish HoustonNet, an extensive GPS network designed for monitoring ground deformation throughout the region. By 2024, the network had grown to include about 265 permanent GPS stations, offering an average spatial resolution of about 10 km by 10 km. To ensure the accuracy of measurements and align GPS data precisely with local ground deformation, the Stable Houston Reference Frame 2020 (Houston20) and the Stable Gulf of Mexico Reference Frame 2020 (GOM20) were introduced for providing stable reference systems at the local and regional scales, respectively (Agudelo et al. 2020; Wang et al. 2020). Further insights into HoustonNet's data products and processing methodologies are available in Wang et al. (2022).

### Interferometric Synthetic Aperture Radar (InSAR)

InSAR is a technique for capturing and analyzing land surface deformation over time and across vast spaces. By examining phase differences in radar signals emitted at different times along the same path, InSAR generates comprehensive subsidence profiles through detailed displacement maps and time series. Urban subsidence researchers have been utilizing InSAR since the 1990s to map subsidence patterns (e.g., Galloway et al. 1999).

Since 1992, a sequence of Synthetic Aperture Radar (SAR) images has been captured over the Greater Houston region using satellites such as ERS‐1/2, Envisat, Advanced Land Observing Satellite (ALOS), and Sentinel‐1. The USGS commenced the application of InSAR for subsidence studies in the Houston region in the early 2000s, enhancing our understanding of subsidence dynamics (Stork and Sneed 2002; Bawden et al. 2012). Additionally, multiple academic research teams have engaged with InSAR data for Houston‐area studies, offering profound insights into the historical and ongoing subsidence trends (e.g., Buckley et al. 2003; Qu et al. 2015, 2019; Miller and Shirzaei 2019; Khan et al. 2022; Liu et al. 2022; Zhong et al. 2022).

### Borehole Extensometers

From the 1970s to the early 1980s, the USGS and HGSD have maintained a network of deep borehole extensometers in the Houston area. This network currently includes 14 extensometers spread across 12 sites, with the most recent installations in Katy, Fort Bend County, in 2017 (Figure 9). These extensometers monitor land surface elevation changes by recording the aquifer compaction between the land surface and the base of each borehole. The extensometers are constructed with a robust double‐pipe design, consisting of an outer steel casing and an inner reference pole. Additionally, each borehole is equipped to measure GWLs within the confined aquifer at the depth of the borehole. A detailed summary of these extensometers can be found in USGS publications (e.g., Gabrysch 1982; Riley 1984; Adams and Ramage 2024). The extensometer datasets have been invaluable to subsidence research, contributing significantly to studies conducted by the USGS, HGSD, and numerous academic researchers (e.g., Wang et al. 2014; Kearns et al. 2015; Liu et al. 2019a).

**Figure 10:**
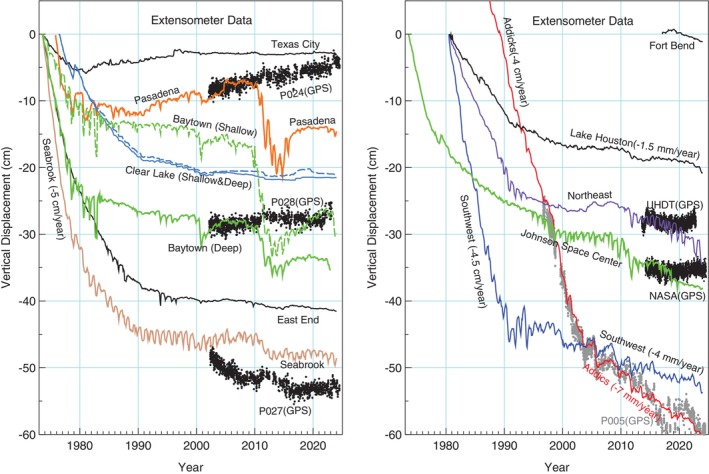
Vertical displacement time series from monthly measurements at 14 extensometers in the Greater Houston region. For comparison, daily vertical displacement time series from GPS stations near the Pasadena, Baytown, Seabrook, Northeast, Johnson Space Center, and Addicks extensometers are also shown. The GPS time series is referenced to the Stable Gulf of Mexico Reference Frame 2020 (GOM20) (Wang et al. 2020). Locations of extensometers and GPS are shown in Figure 9.

Figure 10 presents the time series of aquifer compaction and extension recorded by these extensometers throughout their operational history. Notably, the extensometers in Pasadena and Baytown documented a period of rapid subsidence between 2011 and 2014, which coincided with a prolonged severe drought in southern Texas. Interestingly, this rapid subsidence was not observed in the GPS stations located within the same area. To provide a comparison, the vertical displacement time series recorded by two long‐term GPS stations P024 and P028 (2002‐2023) in the Pasadena–Baytown area are also plotted in Figure 10a. GPS station P024 is located approximately 12 km from the Pasadena extensometer, and GPS station P028 is around 11 km from the Baytown extensometer site. The Baytown site houses both shallow (−131 m) and deep (−450 m) extensometers, spaced about 20 m apart. Locations of the GPS and extensometers are shown in Figure 9.

The rapid displacement observed in the extensometer data at Pasadena and Baytown from 2011 to 2014 may partly be due to site‐specific compaction (both elastic and inelastic) of shallow expansive soils associated with the prolonged severe drought that occurred in southern Texas during the same period. Drought‐induced rapid land subsidence, linked to the compaction of shallow expansive soils, has also been observed in the Galveston coastal area through GPS measurements mounted on the land surface (Welch et al. 2024). Fortunately, since 2015, adjacent GPS data have generally aligned well with the extensometer data at both the Pasadena and Baytown sites. It is plausible that the equipment may not have functioned optimally in response to the rapid compaction and expansion of shallow sediments associated with the prolonged drought from 2011 to 2014. These borehole extensometers have been in the field for around 50 years. Over such a long timeframe, the steel pipes used in these systems can become corroded or bent, which could lead to inaccurate readings. While caution should be exercised when interpreting recent data from Baytown and Pasadena extensometers due to potential equipment issues, the reliability of other extensometers remains high well into the early 2020s (Wang 2023b). For example, GPS station P005, located just about 50 m from the Addicks extensometer, shows consistent agreement with the extensometer data throughout their entire history (1996‐2024) (Figure 10b).

**Figure 11:**
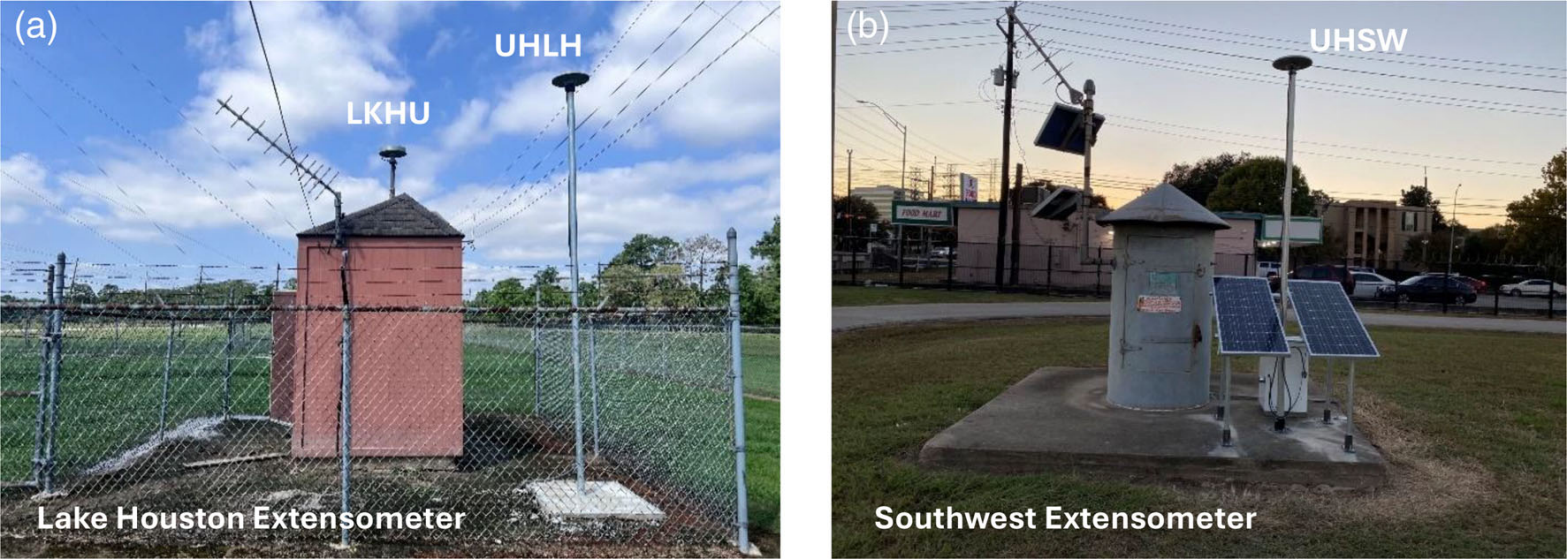
Integrated land subsidence monitoring stations using GPS and extensometers: (a) The Lake Houston extensometer with GPS antenna (LKHU) mounted on the inner pole and GPS (UHLH) mounted on the ground surface. (b) The Southwest extensometer with GPS (UHSW) mounted on a concrete platform supported by steel piles driven six meters deep into the ground.

In response to the potential aging problem of extensometers, HGSD and UH are working together to augment extensometer sites with permanent GPS stations to ensure the continuity of long‐term subsidence monitoring records. As of 2022, seven of the 12 extensometer sites have been equipped with collocated GPS stations. Figure 11 illustrates subsidence monitoring at Lake Houston and Southwest extensometer sites with collocated GPS stations, showcasing the integration of technologies for enhanced monitoring reliability and accuracy.

GPS station P027, located adjacent to the Seabrook extensometer, shows a slightly different displacement time series to the extensometer data over the 2002‐2023 period, although the rate of linear regression would be very similar. Similarly, GPS station NASA, co‐located with the JSC extensometer (Figure 2b), and GPS station UHDT, located just 3.5 km from the Northeast extensometer, exhibit subtle differences in displacement trends when compared to their respective extensometer records. It is important to note that extensometers measure sediment deformation between the land surface and the bottom of the borehole, while GPS units record total displacement below the land surface in general. In the Houston region, GPS antennas are typically mounted on building sidewalls or directly anchored into boreholes several meters deep. For example, all PAM GPS stations operated by HGSD are anchored in boreholes approximately 10 m deep. Similarly, the concrete pad used for supporting the extensometer house, which represents the land surface, is supported by a set of steel piers embedded 6 m into the ground (Gabrysch 1982). This means that both GPS and extensometer data may exclude deformation of the upper several meters of soils, which can experience significant elastic and minor inelastic changes during droughts (Welch et al. 2024), a frequent occurrence in Houston over the past few decades. Therefore, when comparing GPS and extensometer datasets, it is crucial to consider the inherent differences between the two methods and the spatial and temporal variability of land surface deformation.

### Tide Gauges

A tide gauge is equipped with sensors that continuously record the water level surrounding it relative to the fixed land elevation where the device is installed. Differential subsidence between two tide gauge sites can be estimated by comparing their respective water level measurements, assuming the water level changes remain consistent between the two locations. During the 1960s and 1970s, tide gauges, alongside stream gauges, were utilized for land subsidence monitoring along the Houston Ship Channel and Galveston Bay areas. Gabrysch (1982) detailed methods to determine land subsidence at five tide gauge sites within the Galveston Bay complex and two stream gauge sites along the tidal reach of Buffalo Bayou.

In the 2000s, the Center for Operational Oceanographic Products and Services (CO‐OPS) at NOAA operated about 30 tide gauges in the Galveston Bay area (NOAA 2024). As of the early 2020s, many of these gauges have accumulated over 20 years of operational data, providing a promising foundation for detecting land surface elevation changes in the near future. According to Wang (2023c), a 20‐year tide gauge time series can result in an uncertainty of 3‐5 mm/year (95% confidence interval) when estimating the rate of relative sea‐level change, while a 30‐year dataset can reduce this uncertainty to approximately 2 mm/year. To achieve sub‐millimeter accuracy (<1 mm/year), around 60 years of tide gauge observations are required.

**Figure 12:**
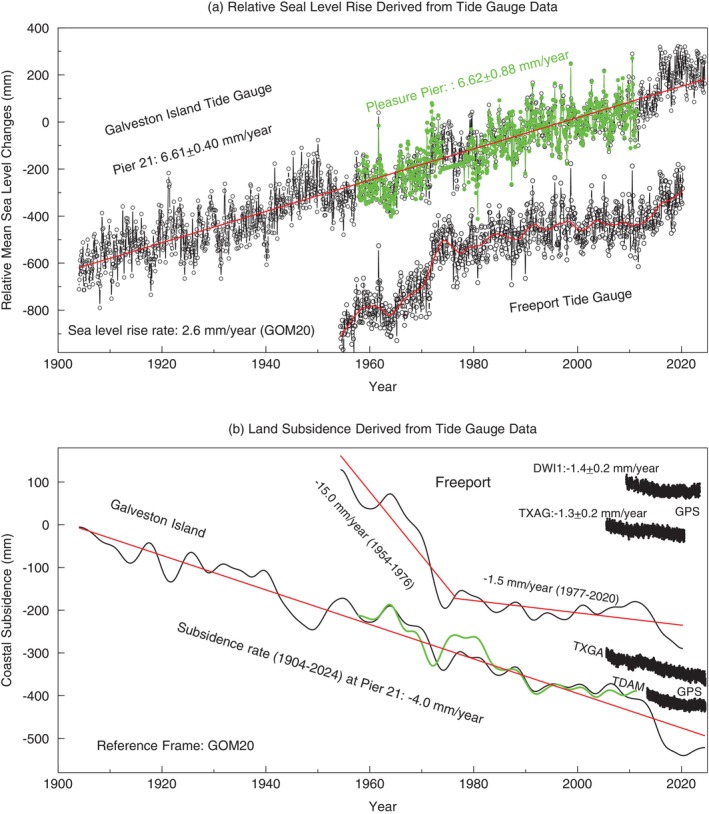
(a) Relative sea‐level changes, or coastal land submergence, derived from tide gauges at Galveston Island (Pier 21, NOAA ID: 8771450; Pleasure Pier, NOAA ID: 8771510) and Freeport (NOAA ID: 8772447), Texas. (b) Coastal subsidence rates calculated by subtracting the average sea‐level rise rate (2.6 mm/year with respect to GOM20) from the relative sea‐level rise rates recorded by tide gauges. The dark lines represent the trend of relative sea‐level changes, smoothed from the tide gauge time series. For comparison, GPS‐derived land subsidence time series (with respect to GOM20) from sites near the tide gauges are also shown. The locations of the tide gauges and GPS are indicated in Figure 9.

There are 11 long‐established tide gauge stations, with over 60 years of data, along the Texas Gulf of Mexico coast (https://www.tidesandcurrents.noaa.gov/sltrends). The longest record spans 120 years (1904‐2024) at Galveston Pier 21. These century‐long datasets have been invaluable in capturing coastal subsidence that modern GPS and inland extensometers did not record. Galveston Island is home to two of these long‐running stations: one at Galveston Pier 21 (1904‐2024) and another at Pleasure Pier (1957‐2011), located about 3 km apart (Figure 9). Both stations recorded an average relative sea‐level rise of 6.6 mm/year over their operational periods (Figure 12a), reflecting the combined effects of sea‐level rise and coastal subsidence. This indicates the rate at which the coastal land is sinking into the ocean. In comparison, the average sea‐level rise rate along the Gulf of Mexico over the past century is around 2.6 mm/year with respect to GOM20, which is tied to the stable inland of the Gulf of Mexico coastal plain (Wang et al. 2020). The century‐long tide gauge data suggest that the average subsidence rate at this location is approximately 4 mm/year with respect to GOM20, a finding supported by long‐term data from GPS stations on the island, such as TXGA (2005‐2024) and TDAM (2014‐2024), which reveal similar rates.

In contrast, the tide gauge at Freeport recorded significant anthropogenic subsidence between 1954 and the mid‐1970s, with an average rate of 15 mm/year, largely due to excessive groundwater pumping for industrial purposes during this period. Leveling surveys indicate that the Freeport area experienced about 70 cm of subsidence from 1906 to 1978 (Gabrysch 1982). Since the mid‐1970s, anthropogenic subsidence has ceased in the Freeport area, and coastal subsidence is now primarily driven by natural subsidence, with a rate of approximately 1.5 mm/year (Figure 12b). Nearby GPS stations, located slightly inland, confirm similar subsidence rates: DWI1 at −1.4 ± 0.2 mm/year and TXAG at −1.3 ± 0.2 mm/year. The uncertainties represent the 95% confidence interval of the estimated subsidence rates (Wang 2022).

### Airborne Light Detection and Ranging (LiDAR) Mapping

LiDAR mapping has proven to be instrumental in generating digital elevation models (DEMs) of bare‐earth surfaces, facilitating the monitoring of land topography changes over time. The first airborne LiDAR datasets specifically covering Harris County were acquired in 2001 by the Harris County Flood Control District, in the aftermath of Tropical Storm Allison in June 2001. Utilizing these datasets, the USGS crafted a land subsidence map for Harris County that spans the years from 1915‐1917 to 2001 (Kasmarek et al. 2009). This involved using Geographic Information System (GIS) techniques to compare the 1915‐1917 land‐surface elevations against the 2001 LiDAR‐derived DEM, providing high‐resolution insights into subsidence patterns across the county and marking a significant leap forward in subsidence monitoring capabilities.

Since the acquisition of the initial datasets in 2001, numerous airborne LiDAR datasets have been collected across the Houston region, greatly enhancing our ability to track topographic changes. These valuable datasets are archived and accessible through the NOAA Digital Coast Program. However, it is imperative to understand that accurately interpreting topographic changes using these datasets requires rigorous data processing and calibration. The feasibility of employing differential LiDAR techniques for ground deformation studies must be thoroughly evaluated to ensure their effectiveness and reliability (Xiong et al. 2018).

## Major Discoveries

### Groundwater Pumping: The Primary Cause of Urban Subsidence

Before the 1960s, the connection between groundwater extraction and subsidence was not immediately evident. Other factors, such as oil and gas extraction and local fault activity, were also considered significant contributors. However, as evidence from GWL measurements grew, and subsidence increasingly led to infrastructure damage and altered drainage patterns, it became clear that groundwater withdrawal was the primary driver of land subsidence. Accordingly, since its establishment in 1975, HGSD has made reducing groundwater pumpage its central task to control subsidence.

Oil and gas development, a key component of the Houston region's economy for over a century, has historically coexisted with groundwater extraction. Shallow oil and gas reservoirs, located between 1000 and 3000 m below the surface, were once associated with both localized and regional subsidence (e.g., Sharp and Hill 1995; Morton et al. 2006). However, since the 2000s, the subsidence impacts of oil and gas activities have significantly diminished. Oil wells now typically reach depths beyond 3000 m, and onshore production has declined. Additionally, since the 1990s, a major shift in oil and gas operations has moved production offshore into the Gulf of Mexico, leaving most onshore reservoirs in their tertiary recovery phase. Enhanced oil recovery (EOR) techniques, such as CO_2_ and saline water reinjection, are now used to maintain reservoir pressures and mitigate subsidence. As a result, the current impact of oil and gas exploration on land subsidence is limited, with only minor, localized subsidence observed in some active oil and gas fields.

Fault movements in the Houston area were also once considered a contributor to subsidence (e.g., Kreitler 1977; Coplin and Galloway 1999). Active faults were first identified in Houston as early as 1926 (Pratt and Johnson 1926), and by 1979, more than 150 historically active faults had been documented, with a combined length of over 500 km (Verbeek et al. 1979; Shah and Lanning‐Rush 2005). In recent years, additional urban faults have been identified using high‐resolution DEMs derived from airborne LiDAR and InSAR data (e.g., Engelkemeir and Khan 2008; Qu et al. 2017).

The role of fault movements in historical subsidence, particularly from the 1950s to the 1980s, has been well studied (e.g., Campbell et al. 2018). Fault activity has significantly declined since the 1990s, coinciding with rising GWLs due to regulatory controls on groundwater withdrawal. This reduction in fault activity and subsidence is supported by fewer instances of fault‐related damage and long‐term GPS data. Accordingly, faulting is now considered a negligible factor in current land subsidence (Liu et al. 2019b; Wang et al. 2022).

These findings on the minor contributions of oil and gas exploration and fault movements to land subsidence have solidified the focus of subsidence management agencies, like HGSD and FBSD, on groundwater withdrawals as the primary cause. This ensures that less significant factors do not divert attention from the core issue.

### Identifying Compacted Aquifer Segments: Vital for Groundwater Withdrawal Optimization

The confined Chicot‐Evangeline aquifer system has been a crucial source of municipal water supply for the Houston‐Galveston area. Initially, water extraction centered on the shallower Chicot aquifer, but over time, the depth of groundwater wells has progressively increased, with recent wells typically extending to depths of 200 to 500 m. Additionally, since the 2000s, the Jasper aquifer has begun to supply significant amounts of groundwater in Montgomery County and northern Harris County, accounting for about half of Montgomery County's groundwater usage (Thornhill and Keester 2020). Efforts to tap into the Catahoula confining unit underlay the Jasper aquifer within Montgomery County have also been underway since the mid‐2010s (Young et al. 2017; Kelley et al. 2018).

A critical consideration for optimizing groundwater pumping is understanding which segments of these aquifers have undergone compaction. Identifying these compacted areas is pivotal for prioritizing resource management and investment strategies, such as determining the optimal depths and locations for production wells to balance urban development with groundwater sustainability.

The Evangeline aquifer extends deeper, lying around 800 m below surface level in downtown Houston, 1300 m in the NASA area, and up to 1700 m in the Galveston coastal region (Ellis et al. 2023). Yu et al. (2014) concluded that by the 2010s, permanent land subsidence in the Southeast Houston area was driven by inelastic aquifer compaction limited to the upper 600 m below the land surface. This conclusion was based on the integration of long‐term extensometer observations, groundwater well measurements, and GPS analyses in the region. This compaction affected both the Chicot aquifer and the upper portion of the Evangeline aquifer, while the lower portion of the Evangeline aquifer showed no considerable inelastic compaction related to groundwater pumping.

Conversely, in Montgomery County, groundwater extraction primarily targets the deeper sections of the Evangeline and Jasper aquifers, with Jasper extractions visibly increasing since the 2000s. Wang et al. (2021) highlighted that compaction in the Jasper aquifer was initiated in the mid‐2000s. Throughout the 2010s, the Jasper aquifer contributed to approximately one‐third of Montgomery County's total subsidence, with the remaining two‐thirds stemming from the Evangeline aquifer. The USGS 2023 Gulf model findings align with these observations (Ellis et al. 2023). These insights are invaluable for formulating effective groundwater management and subsidence mitigation plans.

### Natural Subsidence: A Critical Factor in Coastal Subsidence and Flooding Mitigation

Ongoing subsidence in coastal areas results from a combination of anthropogenic and natural processes. The natural processes include the compaction of unconsolidated sediments under gravity, glacial isostatic adjustment (GIA), and downward tectonic movements associated with the Gulf of Mexico's growth fault system. While GIA and tectonic movements generally occur at regional scales, their impact is relatively minor, typically less than 1 mm/year in the Gulf Coast region (with respect to the Earth's center) (Peltier et al. 2015).

Natural subsidence refers specifically to the geological compaction of sediments due to the natural load of overlying materials on the aquifer system. The Houston‐Galveston coastal area, geologically known as the Houston Embayment, contains thick layers of clays, sands, and gravels that extend over 3000 m deep in the Galveston coastal area, contributing significantly to natural subsidence, the gradual settlement of unconsolidated sediments due to the pressure exerted by the weight of overlying sediments.

It is widely understood that anthropogenic subsidence can vary significantly across time and space but will eventually cease if fluid withdrawals are properly managed. In contrast, natural subsidence will persist over the long term, continuing at a steady rate over centuries, potentially exerting significant impacts on the coastal economy and environment. However, determining the rate of natural subsidence is difficult, as it is often masked by anthropogenic subsidence and other natural events.

Zhou et al. (2021) investigated the rates of natural subsidence along the 600‐km‐long Texas coastal belt. It is found that the rate of natural subsidence in the Galveston coastal area averages 3 to 4 mm/year, while about 1.5 mm/year in the Freeport coastal area, with respect to GOM20 (see Figure 12). This rate decreases slightly inland as sediment thickness diminishes. Since the 2000s, the influence of natural subsidence has become more prominent as anthropogenic subsidence has diminished and concern over sea‐level rise has intensified among the public, researchers, and policymakers. These insights into natural subsidence are crucial for accurately evaluating coastal submergence and flooding risks. They provide valuable guidance for the public and urban planners, helping to inform long‐term mitigation strategies.

One of the major challenges in studying natural subsidence is the inconsistency in reference systems. Over the years, researchers have used different datasets that were tied to various reference systems, resulting in divergent estimates (e.g., Paine 1993; Sharp and Hill 1995; Morton et al. 2001). To address this issue, Wang et al. (2020) developed GOM20, a regional geodetic reference frame realized by long‐term observations (>10 years) at 55 continuous GPS stations located on the stable portion of the Gulf of Mexico Coastal Plain, outside the Gulf coast aquifer covering area. GOM20 enables accurate mapping of natural sediment compaction by excluding the effects of GIA, tectonic movements, and other regional‐scale displacements.

### Consequences of Permanent Subsidence: Groundwater Storage Capacity Loss

Permanent subsidence compresses the pore spaces in the aquifer, leading to a decrease in its capacity to store groundwater. While land subsidence comprises both elastic (recoverable) and inelastic (unrecoverable) compaction of the aquifer system, it is the inelastic compaction that results in irreversible groundwater storage capacity loss. Research indicates that the elastic portion of subsidence accounts for a minimal fraction, less than 10%, of total subsidence in the Houston area, suggesting the majority of subsidence reflects a permanent withdrawal of groundwater from the confined Chicot‐Evangeline system (Holzer and Gabrysch 1987; Wang 2023b). Consequently, groundwater extraction is effectively depleting a finite resource, similar to mining a non‐renewable material for economic gain. This irreversible loss of storage capacity is a critical concern for the region's long‐term water security, as it represents a permanent depletion of a vital resource.

The loss of groundwater storage capacity in confined aquifers can be approximated by assessing the volume of permanent land subsidence (e.g., Smith et al. 2017; Smith and Majumdar 2020; Hasan et al. 2023). In the Houston region, this phenomenon has been meticulously recorded through a series of subsidence contour maps published by the USGS and HGSD, and recent GPS datasets. Building on these studies, we have estimated total land subsidence (1906–2024) at 12 extensometer sites and 220 GPS sites across the Greater Houston region (Figure 1). This estimation utilized contour lines of subsidence from Gabrysch (1982) for 1906–1978, Gabrysch and Neighbors (2005) for 1906–2000, land subsidence data recorded by extensometers from the 1970s to 2024 (Adams and Ramage 2024), and GPS data from the HoustonNet (Wang et al. 2022).

The majority of HoustonNet GPS stations were installed after 2010, and at most sites, subsidence from 2011 to 2024 can be confidently estimated using GPS‐derived vertical displacement time series. The earliest permanent GPS stations (P001, P002, P003, and P004) were established in 1994, and by 2010, approximately 70 permanent stations were in operation. Estimating subsidence between 2001 and 2010 at many GPS sites without data or less than 3‐year data presents certain challenges. For stations with a minimum 3‐year data from this period, a linear trend is calculated to estimate displacement for the entire period (2001‐2010). Additionally, subsidence rates from nearby GPS stations with longer data histories and from extensometers are taken into account. In areas with sparse GPS data during this period, subsidence rates derived from InSAR studies (e.g., Qu et al. 2015) are also used as a reference.

Based on the estimated total subsidence (1906‐2024) at about 230 reference sites, a subsidence contour map was generated, as shown in Figure 1. Considering the natural subsidence (approximately 1 to 2 mm/year in inland area and 2 to 4 mm/year in coastal areas) that has occurred within the unconsolidated sediments over the past 120 years, it is reasonable to define the 0.3‐m (or 1‐ft) contour as the threshold for notable subsidence areas. The area encompassed by the 0.3‐m contour line is approximately 12,000 km^2^, with an average subsidence of about 1 m and a total subsidence volume of about 12 km^3^. Approximately 65% of this volume occurred between 1906 and 1978 (7.8 km^3^), before the establishment of the HGSD, 20% between 1979 and 2000 (2.4 km^3^), and about 15% is attributed to more recent subsidence from 2001 to 2024 (1.8 km^3^).

The volume of total land subsidence directly correlates with the loss of groundwater storage capacity. To contextualize this loss, it can be compared to the storage capacity of Lake Houston, a significant reservoir and a primary source of drinking water for the city. Lake Houston has a storage capacity of approximately 160,000 acre‐feet, which is equivalent to about 0.2 km^3^. The groundwater storage capacity loss of 12 km^3^ due to permanent aquifer compaction in the Houston area from 1906 to 2024 is roughly equivalent to 60 times the volume of Lake Houston. The total water use for Harris and Galveston counties in 2023 is 1077.7 MGD, which amounts to roughly 1.5 km^3^ over the entire year. To put that into perspective, the lost groundwater storage capacity is equivalent to approximately 8 years' worth of water usage at the current rate for these two counties.

The substantial loss of land surface elevation and groundwater storage capacity carries several significant negative consequences. One of the most immediate effects is the increased vulnerability to flooding. As land subsides unevenly over space, it causes changes in the land surface that can overwhelm drainage systems and exacerbate flood risks. Additionally, the diminished groundwater storage capacity reduces the region's resilience to drought and other emergencies. Groundwater is a crucial backup supply for maintaining water security in large cities, and with decreased storage, the risk of water shortages grows, potentially impacting agricultural productivity, industrial operations, and daily life for residents.

Moreover, the rapid reduction in groundwater storage can lead to long‐term environmental degradation. Subsidence can disrupt natural drainage patterns, damage ecosystems, and diminish the effectiveness of wetlands, which serve as natural buffers against floods and provide essential habitats for wildlife. The loss of these natural functions can have cascading effects on biodiversity and the overall health of the environment.

### Climate Change: Posing Profound Threats to Subsidence and Groundwater Management

Groundwater is often a major water source for large urban areas and is consistently relied upon as a dependable emergency backup for freshwater. As climate change leads to more frequent and severe droughts, dependence on groundwater extraction is likely to increase during these periods. This pattern was evident during recent droughts in Southern Texas, including those in 2011, 2018, 2020, 2022, and 2023 (see water use data in Figures 5 and 6). The intensified pumping during these events could potentially cause a resurgence of subsidence in areas where it had previously been controlled or worsen existing subsidence problems.

The Texas coastal area, particularly the Houston‐Galveston region, is underlain by the Beaumont Formation, known as expansive soils for its high smectite content, with thickness varying from a few meters to over 100 m depending on the location (Stoeser et al. 2005). Recent research by Welch et al. (2024) has shown that drought events during the summers of 2018, 2020, 2022, and 2023 have resulted in step‐like inelastic compaction in the expansive soils of the Galveston coastal area. While each drought event individually contributes to minor inelastic compaction on the order of a few millimeters—amounting to approximately 10% of the total compaction in the shallow soils during each drought—the cumulative effect of repeated droughts could lead to profound permanent land subsidence over time, a risk previously underappreciated in coastal subsidence and relative sea‐level rise studies.

Climate change exacerbates both coastal subsidence and sea level rise, resulting in accelerated coastal submergence and posing a significant threat to coastal infrastructure and ecosystems. This synergistic effect intensifies the challenges faced by coastal communities. In response, HGSD, FBSD, and GCDs, in collaboration with local stakeholders, are actively working to develop resilient communities capable of addressing the complex interplay between climate change and land subsidence.

### New Pre‐Consolidation Head (NPCH) and Safe Pumping Buffer (SPB): The Thresholds for Preventing Subsidence to Reoccur

As we progress into the 2020s, only about 1500 km^2^—approximately one‐twentieth of the Greater Houston region—continues to experience moderate subsidence rates of 1 to 3 cm/year (see Figure 9). In other parts of Houston, subsidence rates are either insignificant (5 to 10 mm/year) or have nearly ceased (<5 mm/year). Groundwater serves as a critical emergency freshwater reserve during droughts, infrastructure failures, or surface water contamination, necessitating measures to prevent land subsidence while ensuring sustainable use. In the summer of 2023, the Greater Houston region experienced a D4 drought, among the most severe in its history, significantly stressing groundwater resources in the region. The D4 level, classified as Exceptional Drought by the U.S. Drought Monitor, indicates extreme water shortages, widespread crop and pasture losses, and severe economic impacts (USDM 2023).

In response to the rapidly rising demand for groundwater, the LSGCD, which oversees groundwater in Montgomery County, implemented a Temporary Drought Buffer in mid‐August 2023. This buffer retroactively increased the Annual Production Limitations by 10% for the entire year. By mid‐September, more than 40% of Montgomery County was experiencing Exceptional Drought conditions, prompting LSGCD to raise the buffer further to 15% (LSGCD 2023). This escalation in groundwater pumping raised concerns about exacerbating the already significant subsidence issues in southern Montgomery County, where substantial infrastructure and building damage have occurred over the past two decades. The lowest sustainable GWLs in the primary aquifers (Evangeline, and Jasper), as well as the maximum groundwater extraction volume that can be maintained without causing rapid subsidence, are currently unknown at the administrative level.

Wang (2023a) introduced the concept of NPCH, identifying it as a crucial threshold in subsidence management. The NPCH represents the lowest hydraulic head within the clay layers of the confined aquifer during a subsidence cycle and is determined by the GWL at the point when land rebound begins. Maintaining GWLs above this threshold ensures that extraction does not cause significant inelastic compaction, thereby averting permanent subsidence. Building on this, Wang et al. (2024) introduced the SPB concept, which quantifies the difference between the current GWL and the NPCH in areas where subsidence had ceased, indicating the maximum allowable groundwater‐level drop before permanent subsidence resumes. SPB is instrumental in determining the maximal volume of groundwater that can be safely extracted without risking permanent subsidence.

The NPCH and SPB serve as critical benchmarks for sustainable groundwater management, providing policymakers with clear guidelines for setting safe extraction limits to prevent the recurrence of land subsidence in areas where it has been mitigated. By incorporating these innovative concepts into groundwater management strategies, the region can ensure a more resilient and sustainable future.

### Subsidence Modeling: A Key Tool for Long‐Term Groundwater Planning and Subsidence Risk Assessment

Over recent decades, a comprehensive suite of 10 publicly documented groundwater modeling studies in the Greater Houston region has significantly advanced our understanding and management of groundwater and subsidence. Early modeling efforts, such as Wood and Gabrysch (1965), utilized an electric‐analog method to emulate the Chicot and Evangeline aquifers' groundwater flow, emphasizing the implications of intensive aquifer pumping on GWLs. Following studies, including those by Jorgensen (1975) and Meyer and Carr (1979), enhanced spatial coverage and adopted finite‐difference (FD) coding, offering more intricate simulations of hydrogeologic units and incorporating subsidence predictions based on aquifer compaction and fine‐grained sediment storage alterations.

In 1985, the USGS expanded its scope with regional models covering areas from Louisiana to near Mexico, focusing on GWL responses and subsidence (Carr et al. 1985). Building on this foundation, significant advancements were achieved through the USGS Regional Aquifer‐System Analysis Program, which utilized the Kuiper code to extensively model groundwater flow across the Gulf Coast aquifer system and surrounding regions (Williamson et al. 1990; Ryder and Ardis 2002).

Entering the 2000s, the USGS, alongside HGSD, FBSD, and additional local collaborators, embarked on a series of advanced MODFLOW‐based modeling efforts. Kasmarek and Strom (2002), integrated the Interbed‐Storage package with MODFLOW to simulate changes in aquifer‐system compaction and storage (Kasmarek and Strom 2002). Kasmarek and Robinson, in 2004, introduced the Northern Gulf Coast Groundwater Availability Model (NGC‐GAM), employing a head‐based approach to replicate subsidence within the Chicot and Evangeline aquifers (Kasmarek and Robinson 2004). Further refining these models, Kasmarek in 2012 unveiled the Houston Area Groundwater Model (HAGM), updating the NGC‐GAM and incorporating a cutting‐edge subsidence code to model compaction across various aquifers and confining units (Kasmarek 2012). The latest iteration, the GULF‐2023, developed by USGS, merges extensive groundwater‐level and subsidence data from the Greater Houston region, marking a significant leap in modeling accuracy and applicability (Ellis et al. 2023).

This ongoing evolution of modeling studies, underpinned by the growing pool of GWL and subsidence data, is crucial for continuously refining the region's regulatory measures in tune with changing environmental conditions and technological progress. Groundwater and subsidence models transcend their academic origins, playing an instrumental role in practical water resource management and land subsidence mitigation. By mapping out the potential impacts of different groundwater use scenarios, these models offer invaluable insights for crafting effective regulatory and conservation strategies, ensuring the water resources' sustainable use and protecting land surfaces from the adverse effects of extensive groundwater extraction.

## Future Directions and Sustainability

### Advancing Monitoring Technologies and Strategies

The long‐term sustainability of groundwater and subsidence management in the Houston region calls for a proactive, forward‐thinking approach centered on continuous, precise, and near real‐time monitoring systems. Collaborative efforts between USGS, HGSD, and FBSD have been foundational in advancing GWL monitoring initiatives. By the early 2020s, approximately 650 active wells were monitoring GWLs across the primary aquifers (e.g., Ramage and Braun 2023; Ramage 2024). However, the predominance of these wells still depends on manual data retrieval, often occurring only a few times a year, which underscores the urgent need for enhancing our monitoring capabilities to real‐time or near real‐time systems.

In the realm of subsidence monitoring, HGSD, FBSD, and UH have been at the forefront, adopting state‐of‐the‐art satellite technologies. The deployment of the dense GPS network since the mid‐1990s has markedly advanced our capacity to monitor subsidence over wide areas. Despite its extensive coverage, certain areas, particularly in the northwestern and northern regions currently experiencing moderate subsidence (>1 cm/year, see Figure 9), require a more detailed understanding of subsidence patterns. To this end, the fusion of Synthetic Aperture Radar (SAR) data with GPS data has commenced, enriching our comprehension of the rates and patterns of subsidence. Since 2019, a partnership between HGSD, FBSD, and Southern Methodist University (SMU) has enabled the integration of InSAR into regular subsidence monitoring workflows.

Further work by researchers at UH has led to the development of methodologies that combine GPS and Sentinel‐1 InSAR data for near‐real‐time subsidence monitoring across the broader Houston region (Liu et al. 2022). By leveraging groundwater‐level data, GPS data, satellite imagery, and advanced computing technologies, efforts are underway to create an integrated GPS and InSAR automated subsidence monitoring system with near‐real‐time capabilities. This system would enable more proactive decision‐making and swift action, enhancing emergency responses as the frequency of droughts increases due to climate change.

### Exploring Surface Water Alternatives and Promoting Water Conservation

As Houston's population continues to grow, the increasing demand for water underscores the urgent need for sustainable water sources that extend beyond the current groundwater and surface water capacities. To address this, HGSD, FBSD, and GCDs are intensifying efforts in water conservation and exploring alternative water solutions, actively engaging local stakeholders in these initiatives. Historically, the construction of reservoirs along the San Jacinto, Trinity, and Brazos River Basins, beginning in the 1950s, has been instrumental in meeting the region's water needs. This effort has grown to encompass numerous water treatment plants, including those operated by the City of Houston, City of Sugar Land, City of Richmond, the Gulf Coast Water Authority, the Brazosport Water Authority, and others, all of which now play a vital role in the region's water infrastructure.

In the early 2020s, several large‐scale groundwater distribution and supply projects are underway, including the Luce Bayou Interbasin Transfer Project (LBITP), the Northeast Water Purification Plant Expansion (NEWPP) project, the Northeast Transmission Line (NETL) project, and the Surface Water Supply Project (SWSP) (Petersen et al. 2020). Simultaneously, HGSD and FBSD are exploring innovative water management strategies such as recycled water, desalination, and artificial groundwater recharge. Collaborative initiatives assessing the feasibility of seawater desalination and managed aquifer recharge signal the emergence of next‐generation water supply solutions. By promoting green infrastructure and pioneering water sourcing strategies, Houston is positioning itself to enhance environmental resilience and mitigate subsidence risks, laying a critical foundation for the sustainable management of water resources in the area.

## Summary and Conclusions

The prolonged engagement with land subsidence over the past century in the Houston region has shed light on its predominantly human‐induced nature, challenging the misconception of it being a “natural” hazard. This experience has critically emphasized the importance of robust groundwater regulations in effectively managing subsidence. Thanks to astute regulatory strategies, local authorities have not only arrested the rapid progression of subsidence but also supported the swift economic and societal growth of the region.

The Houston area's experiences over the past century underscore the complex challenge of managing groundwater within a region characterized by dense population and vigorous economic activity. It highlights the necessity of a nuanced approach that simultaneously caters to immediate community necessities and ensures the long‐term sustainability of groundwater supplies. The cooperation of various stakeholders, encompassing local governments, regulatory agencies, industries, and the community at large, is pivotal in forging a comprehensive strategy to mitigate subsidence issues while promoting sustainable development.

The invaluable lessons gleaned from this extensive period serve as a foundational framework for ongoing scientific research and policy formulation regarding land subsidence. They offer critical insights applicable not only to the Houston region but also to other areas worldwide facing akin challenges, guiding efforts toward a more balanced and sustainable management of subsidence and water resources.

## Authors' Note

The authors do not have any conflicts of interest or financial disclosures to report.

## Data Availability

The data that support the findings of this study are available in USGS Groundwater Data for the Nation at https://waterdata.usgs.gov/nwis/gw. These data were derived from the following resources available in the public domain: NWIS, https://waterdata.usgs.gov/nwis/gw.
